# Innovations in public health surveillance: An overview of novel use of data and analytic methods

**DOI:** 10.14745/ccdr.v50i34a02

**Published:** 2024-04-30

**Authors:** Heather Rilkoff, Shannon Struck, Chelsea Ziegler, Laura Faye, Dana Paquette, David Buckeridge

**Affiliations:** 1 Data, Surveillance and Foresight Branch, Public Health Agency of Canada, Toronto, ON; 2 Data, Surveillance and Foresight Branch, Public Health Agency of Canada, Winnipeg, MB; 3 Data, Surveillance and Foresight Branch, Public Health Agency of Canada, Calgary, AB; 4 Data, Surveillance and Foresight Branch, Public Health Agency of Canada, Ottawa, ON; 5 Data, Surveillance and Foresight Branch, Public Health Agency of Canada, Montréal, QC; 6 School of Population and Global Health, Department of Epidemiology and Biostatistics, McGill University, Montréal, QC

**Keywords:** public health surveillance, innovative methods, novel data, artificial intelligence, wastewater surveillance, nowcasting

## Abstract

Innovative data sources and methods for public health surveillance (PHS) have evolved rapidly over the past 10 years, suggesting the need for a closer look at the scientific maturity, feasibility, and utility of use in real-world situations. This article provides an overview of recent innovations in PHS, including data from social media, internet search engines, the Internet of Things (IoT), wastewater surveillance, participatory surveillance, artificial intelligence (AI), and nowcasting.

Examples identified suggest that novel data sources and analytic methods have the potential to strengthen PHS by improving disease estimates, promoting early warning for disease outbreaks, and generating additional and/or more timely information for public health action. For example, wastewater surveillance has re-emerged as a practical tool for early detection of the coronavirus disease 2019 (COVID-19) and other pathogens, and AI is increasingly used to process large amounts of digital data. Challenges to implementing novel methods include lack of scientific maturity, limited examples of implementation in real-world public health settings, privacy and security risks, and health equity implications. Improving data governance, developing clear policies for the use of AI technologies, and public health workforce development are important next steps towards advancing the use of innovation in PHS.

## Introduction

Public health surveillance (PHS) is the ongoing, systematic collection, analysis, and interpretation of data, followed by the dissemination of information, for the purpose of guiding actions to prevent and control diseases or improve population health ((1–3)). Traditionally, PHS was conducted with a limited number of data sources from public health information systems, health care, and laboratory information systems, as well as questionnaire-based surveys, which often require substantial resources and time to process, analyze, and disseminate.

The digitization of health care and other sectors has reduced the time lag, cost and burden associated with conducting PHS, and enabled exploration of other sources of data to augment traditional sources ((4)). In addition, artificial intelligence (AI) has seen major advances over the past decade. Artificial intelligence-enabled methodologies that efficiently process large amounts of structured and unstructured data are increasingly used in PHS ((5–7)).

Many of these data sources and AI methods were used during the coronavirus disease 2019 (COVID-19) pandemic, where timely and complete information was crucial to understanding and responding to evolving pandemic risks (4). The rapid development of these innovative surveillance methods and use of novel data sources suggests the need to take a closer look at the scientific maturity, as well as the feasibility and utility of their use, in real-world applications ((5,6,8)). The objective of this paper is to highlight examples of the application of innovative methods to PHS and provide insights for public health authorities on the potential benefits, risks, and challenges of using non-traditional data sources and methods in PHS.

This article provides an overview of PHS innovations in data and analytic methods published in the past five years, including any evidence of their application to real-world settings, ethical issues, and known health equity implications. Each innovation is described, including its level of scientific maturity and, where available, any evidence of its impact on surveillance practice or public health action. The results section starts by exploring novel data sources that have been applied to PHS, highlighting successful examples of their application to provide timely, accurate and reliable information to support public health action. It then focuses on innovative methods that have been developed to analyze surveillance data, including the development of AI to support the integration and analysis of large and/or non-traditional data sources and the application of advanced analytic methods to improve nowcasting of information.

## Methods

### Approach

This overview defines the term “innovative surveillance” broadly as the use of non-traditional data sources and/or analytic methods to detect and understand health events and determinants. The primary focus was on data sources and analytic methods; this overview does not provide detailed discussion of other components of the surveillance process (e.g., dissemination or evaluation strategies).

Relevant topic areas were identified for inclusion in this article by searching PubMed, Embase, Global Health, and Scopus in the spring of 2023. A detailed search strategy, developed with the support of a librarian, was restricted to peer reviewed articles published between January 1, 2013, and February 23, 2023, from member countries of the Organisation for Economic Co-operation and Development (OECD) and China, in English language only. Hand searching provided additional sources.

Results of the literature search were screened for relevance via title and abstract search and grouped into topic areas. Final selection of articles within each topic area was restricted to the past five years (January 1, 2018, to February 23, 2023) to ensure that articles were more reflective of current technological and methodological innovations. As the search yielded a large number of articles on analytic methods, decisions were made by the research team to exclude certain broad analytic topic areas (such as innovations in biostatistics, laboratory, or geospatial analytic methods), and focus on nowcasting and artificial intelligence, two areas that have been adopted by public health from other disciplines.

The authors focused this overview on a subset of articles that met the definition of “innovative surveillance”, discussed steps taken to evaluate or validate the method or data source(s), described potential or actual improvements to the PHS system, and, where possible, showed application to real-world public health practice.

## Results

### Novel data sources and their applications

#### Overview of novel data sources

The rise of digital technologies has made new data sources available for disease surveillance. Commonly used digital data sources include social media and aggregate search query data, where initial surveillance applications date from the early 2000s, as well as participatory surveillance methods, such as repeated cross-sectional online surveys and crowdsourcing of photos or sample submissions ((9)). More recently, PHS applications of other digital technologies are being explored, such as mobility data and the Internet of Things (IoT), which includes wearable devices and other physical objects that connect and exchange data via the Internet ((8)). Digital data sources may have the potential to provide more timely information and capture populations that may not seek health care; although possible to use as an independent source of information, they are generally considered to be complementary to traditional surveillance data ((9)).

#### Social media and web-search data

Social media (e.g., Twitter/X) and web search (e.g., Google Trends) data have been used to support disease surveillance as a source of data for nowcasting, situational awareness, and outbreak detection ((9)). A recent systematic review focusing on communicable disease surveillance noted that the majority of included studies used data from Twitter/X, and that studies that used Twitter/X data showed higher overall reliability and validity than studies using data from other social media platforms ((10)). The review also noted that the majority of studies focused on influenza surveillance, and that additional research was needed to assess the effectiveness of social media for other disease areas ((10)). Other examples of the use of social media and/or web search data included retrospective analyses to evaluate the potential of these sources to predict cases of sexually transmitted and blood-borne infections (STBBIs) ((11)), prioritizing restaurant inspections based on foodborne outbreak information ((12)), drug utilization estimates ((13)), and early warning systems for e-cigarette/vaping-related lung injuries ((14) and COVID-19 outbreaks ((15)).

One of the challenges with the use of digital media is the need to collect and process large quantities of information, either through manual monitoring or automation ((16)). The European Centre for Disease Prevention and Control (ECDC) released epitweetr, an R-based software library that collects, aggregates, detects, and disseminates information for early detection of public health threats using Twitter/X. An evaluation of the tool noted greater timeliness when compared to manual review ((16)). Artificial intelligence methods such as natural language processing, described later in this paper, are also increasingly being used to process and analyze digital information sources.

While the utility of social media and web search data for disease surveillance has been explored for nearly two decades, the validity, reliability, and stability of these data continue to present challenges to developing standardized approaches to using this information ((9)). For example, changes to the query algorithms of search engines, the use of different language styles, confounding search terms, and demographic biases in terms of who uses digital technologies, may impact the quality of information from these sources for PHS ((9,17)). A recent systematic scoping review also noted that most studies on digital surveillance did not utilize their results for public health action, and that more rigorous methods were needed to operationalize this information for public health decision-making ((17)). Surveillance platforms that combine social media, web search, and healthcare data may improve the accuracy of results ((9,18)).

#### Participatory surveillance data

Participatory surveillance involves the voluntary recruitment and engagement of members of the public to participate in repeated surveys or other crowdsourcing methods ((9)). This approach is sometimes used as an augment to traditional disease surveillance, to capture information in a timelier way, and to capture populations that may not seek health care for testing and diagnosis ((8)). Examples include Flu Near You in the United States, InfluenzaNet in Europe ((9)), and FluWatchers in Canada ((19)). Community surveillance using self-collected specimens has also been implemented and has enabled rapid assessment of community-level burden of influenza ((20)). Additionally, studies have explored participatory syndromic surveillance using social media and newspaper reports as a source of information during the COVID-19 pandemic that may be timelier and more accessible than official public health case reports ((21,22)).

Outside of respiratory pathogens, recent studies suggest current use of participatory approaches to support surveillance of potential disease carrying vectors or vector-borne disease. For example, platforms such as iNaturalist, eTick.ca, and Mosquito Alert use crowdsourced photos to identify the distribution and seasonal trends of specific species of ticks and mosquitos ((23–26)), and initiatives such as tickMAP in New York state used community-submitted tick specimens to track the emergence of tick-borne pathogens in near real time ((27)).

Participatory surveillance may be applied in a way that enables participation from equity-deserving populations that may otherwise be excluded from traditional surveillance systems. For example, in a rural Appalachian community, participatory surveillance via an online or phone-based symptom self-checking tool was used to identify at-risk individuals who may otherwise have not sought health care and link them to resources from the local health department ((28)). However, certain populations may be less likely to participate in participatory surveillance, including males, younger and older age groups ((29)), and those with lower income and education ((9)). This may introduce bias and potential health equity issues, particularly if groups that are more likely to experience illness are excluded.

#### New digital data sources

The use of digital data sources, such as mobile technologies, IoT and wearables, represent emerging areas for further exploration. For example, mobility data was used to explore the impact of COVID-19 and government policy on travel patterns. Health inequities were also noted, as socially disadvantaged populations were often unable to benefit from stay-at-home orders ((30,31)).

Wearable devices, such as smartwatches, have been used to collect individual-level data on variables linked to viral infection, such as resting heart rate, sleep, and mobility ((32,33)). As an example, a study noted that wearable technologies may improve nowcasting of influenza-like illness (ILI) rates in the United States ((33)). Various applications of IoT have emerged in the past few years. In one study, researchers placed thermal sensors and microphones in hospital waiting rooms to monitor coughing, which was then used to support ILI surveillance ((34)).

New digital data sources from mobility, wearables, and IoT represent an emerging field that requires greater evaluation and assessment ((8,32)), including careful consideration of privacy and ethical concerns ((35)). Like other digital data sources, these sources involve self-selected populations and exclude groups who do not have access to digital technologies. Privacy issues have also emerged with the use of new digital technologies and social media data; data ownership and the right to share data and use the data for secondary purposes may differ among the public sector (e.g., government), private sector (e.g., Twitter/X), and geopolitical jurisdictions ((9,25)). The need for upgraded infrastructure and investment to support the integration and analysis of information generated from new technologies may also present substantial barriers ((8,36)).

#### Wastewater

Wastewater surveillance (WWS) has evolved as a data source that now supports global surveillance of infectious diseases in a manner that is independent of health-seeking behaviour and healthcare system access ((37,38)). When coupled with small area socio-demographic data, WWS has the potential to forewarn and confirm clinical trends, address health inequities, fill reporting gaps due to waning clinical testing, and provide purpose-built sentinel surveillance of communities with higher-risk profiles for specific pathogens ((38–42)). The deluge of WWS data during the COVID-19 pandemic led to novel analytic methods to help inform public health action. These include sophisticated machine learning algorithms that were applied to estimate sewage flow rates to allow for data normalization ((43)), and the application of simple statistical methods that were then tested to identify early warning signals in a user-friendly manner ((44,45)). New methods developed for WWS during the pandemic were validated by comparing wastewater signals to clinical case data and COVID-like illness syndromic data ((38,40,41,45,46)). Innovations in WWS have also benefited from other novel data linkages. In a recent study in Iceland, wastewater signals were compared with driving under the influence records to help distinguish trends of recreational drug use from increased drug dependencies, the latter of which may require enhanced public health action ((47)).

Wastewater surveillance of COVID-19, other infectious pathogens, and illicit substances, has identified limitations of this approach including the inability to distinguish reasons for signal increases/decreases, the degradation of the pathogen/substance in the wastewater before testing is performed, changing population denominators, and non-standardized sampling methods ((47–49)). Wastewater surveillance is also limited by the epidemiological indicators it can provide (i.e., incidence and prevalence) and the population it can monitor (e.g., includes only those in the sewer shed of a wastewater treatment facility) from the WWS data alone ((45–49)).

### Innovative analytic methods

#### Artificial intelligence

Artificial intelligence, which includes natural language processing (NLP), machine learning, and deep learning, can integrate, process, and interpret multiple sources of information more efficiently and more consistently than humans ((50)). The recent growth in the use of AI-based technologies that can process unstructured text data has enabled the use of novel data sources, including those discussed in the previous sections, to be leveraged more effectively ((7)). Artificial intelligence has enormous potential to improve PHS, as it is capable of processing large amounts of data to identify anomalies that may pose a threat to public health ((7)), however, it is still an emerging field in which more real-world evaluations are needed. Some of the published innovations using AI for PHS still reside within academic collaborations. One such study from the Yale School of Medicine used NLP, which applies AI methods to the interpretation of human language, to provide real-time monitoring of population health by identifying symptoms mentioned on social media platforms ((51)).

Machine learning identifies complex patterns in data for classification and prediction ((50)). In New York City (NYC), machine learning, in combination with NLP, was tested to improve “pre-syndromic surveillance”, which seeks to identify rare or previously unseen threats to health from clinical information ((52)). In this study, multidimensional semantic scan (MUSES) is a machine learning and NLP-based method developed to improve early detection of illness by eliminating the need for predefined case definitions and automatically clustering information by small geographies and/or demographics. MUSES was applied to historical free-text complaint data from NYC emergency departments and was found to identify more events of public health interest and a lower false positive rate than the current approaches used by the New York City Department of Health and Mental Hygiene ((52)). Natural language processing-based PHS has also been tested to improve the timeliness of overdose mortality reporting by eliminating the need for manual coding of free-text death certificates ((53)). The above examples show the potential of AI in PHS, but it remains unclear how many AI methods have been implemented into PHS. One real-world application by the Department of Veterans Affairs in the United States showed successful adaptation of an existing NLP-based PHS method early in the COVID-19 pandemic to monitor travel history in clinical records for public health follow-up (54).

Deep learning is a specialized type of machine learning that incorporates sophisticated neural networks that support classification using large amounts of text and are designed to work in a manner similar to a human brain. It has been increasingly used to support disease surveillance ((7,55)). The Centers for Disease Control and Prevention (CDC) tested neural networks and found that deep learning can interpret physician records to accurately predict the chief complaint, and potentially improve the timeliness and accuracy of information available for syndromic surveillance ((56)). Deep learning has also been applied to internet-based surveillance systems to support early warning, situational awareness, and nowcasting of infectious diseases. For example, Sentinel, an American surveillance system, uses deep learning to identify and classify health-related social media posts, news media, and CDC data to detect possible outbreaks and provide situational awareness ((55)).

The use of AI to support PHS is a new and emerging field that still needs evaluation of implementation into existing public health systems. Algorithms and machine learning models built with inaccurate, incomplete, or unrepresentative datasets, may both limit the accuracy of AI-based methods as well as bias results based on race, gender, or other characteristics ((50,57)). It is important to ensure that there is transparency in how AI models are built so that results are explainable, and that those who are interpreting the outputs of AI analyses are adequately skilled in PHS and can apply appropriate judgment. It is also important for public health professionals to understand AI methods, their applications, and their risks before applying it to public health practice ((57)).

#### Nowcasting

Nowcasting uses recent surveillance data to model the current situation (e.g., case counts) when real-time data are unavailable ((58)). In one study, nowcasting using a Bayesian approach accurately estimated COVID-19 rates to inform resource allocation in NYC, successfully overcoming delays between testing and reporting ((59)). Advances in nowcasting have also been adopted in One Health surveillance systems to help fill data gaps and help anticipate zoonotic outbreaks. For example, the Norwegian Institute of Public Health successfully applied nowcasting principals to respond to gastrointestinal illness outbreaks using *Campylobacter* testing data from poultry farms and meteorological data ((60)). While nowcasting can be useful to estimate current situational awareness during rapidly changing public health emergencies, it is limited by the quality of data and the clarity of the interpretations provided to decision makers ((59,61)).

## Discussion

This review has explored innovations in PHS over the past decade and, where possible, described examples of their applications to PHS programs. Examples of the use of these novel sources to support PHS include providing novel information that improves estimates of disease, promoting early warning and identification of potential threats to health, and generating new information for public health action.

Despite these opportunities, there are substantial challenges to integrating innovations in PHS into practice. As new data sources and methods are added to the PHS toolbox, their risks and benefits should be considered with the goal of improving overall population health. Most of the areas explored in this paper are lacking in scientific maturity, and in many cases, are so novel that standard methods and best practices do not yet exist to help advance these fields reliably and responsibly ((49,50,57)). Many of the novel methods identified in this paper were tested in academic environments with no clear real-life implementation strategy ((51,55)). More evaluations of these interventions in real-world settings, which assess their utility in improving PHS and implications for public health action, are needed. These evaluations could be used to develop and disseminate guidance and standardized approaches to support public health organizations in implementing novel methods.

The use of digital technologies and AI in PHS also introduces challenges for privacy and security, data governance, and ethical considerations. For example, there is a need to balance between the benefits of having large quantities of granular information for analysis and the need to ensure individuals cannot be (re)identified. This is particularly true with AI methods, given the large quantity of information that is usually required to train the model ((54,57,62,63)). In the case of digital data, which may be publicly available, but where permission to use for surveillance purposes has not been acquired, it is not clear how/whether informed consent can or needs to be obtained. Particular care needs to be taken to ensure that data are anonymized and confidential information is not revealed ((63)). Protection of digital data and transparency in how and what data is acquired, stored, and used are key to maintaining public trust and ensuring the sustainability of these systems ((57,64)), and thus progress towards digital data governance is needed to fully operationalize these data sources. Ethical frameworks for the use of AI and social media data in research ((63)), and guidelines for the use of AI more broadly ((65–67)), have been developed to support responsible conduct and protection of individuals from whom data is collected.

Health equity is an important consideration in implementing new surveillance methods. This overview identified several examples of approaches that could be used to support health equity, as they include populations that may be missed in traditional surveillance. However, a recent review article noted that there were no studies that specifically focused on vulnerable populations in the use of digital PHS, and thus substantial work is needed to explore the health equity implications of its use ((17)). Furthermore, greater work is needed to explore, identify, and address biases in AI algorithms and in the data used to train AI algorithms to ensure that these methods are not perpetuating harmful outputs as a consequence of biased inputs ((57)).

## Limitations

Limitations of this overview should be noted. This article was intended to provide a snapshot of recent innovations in PHS and explore examples of real-world application. As such, it is not intended to be an exhaustive list, and cannot provide detailed appraisal of the effectiveness of these innovations. The article focused on peer-reviewed literature only, and thus may have omitted articles from applied public health settings that were published as grey literature. The use of peer-reviewed literature may also have produced a positive publication bias, with studies noting negative results or unintended consequences potentially being under-represented. This is an important consideration given that non-traditional data sources may also be a source of public health misinformation ((68)), and thus require careful consideration and evaluation prior to use.

## Conclusion

Novel data and methods for PHS have the potential to improve the quantity, accuracy, completeness, timeliness, and accessibility of information available for public health response; however, the evidence base to support their utility in the real-world, as opposed to academic, settings appears to be lacking. Substantial barriers prevent the implementation of novel data and methods in PHS, ranging from health equity, privacy, and ethical concerns to training and availability of data and technologies. Improving data governance mechanisms, developing clear policies for ethical use of AI technologies in PHS, and training the public health workforce on the responsible use of innovative technologies are important next steps towards advancing greater use of novel methods and data sources.
